# Caregivers’ knowledge, attitudes, and practices regarding household product safety and poisoning prevention in children in Saudi Arabia

**DOI:** 10.3389/fpubh.2026.1937239

**Published:** 2026-10-19

**Authors:** Hanouf S. Bafhaid

**Affiliations:** Department of Pharmacology and Toxicology, College of Pharmacy, Umm Al-Qura University, Makkah, Saudi Arabia

**Keywords:** caregiver risk management, childhood poisoning, household cleaners, over-the-counter drugs, unintentional household poisoning

## Abstract

**Introduction:**

Household product poisoning is a common cause of emergency department visits and hospital admissions among children. Global variations reflect socioeconomic disparities and the presence of other contributing factors. This study evaluated caregivers’ knowledge, attitude, and practice (KAP) regarding household product safety and childhood poisoning management.

**Methods:**

A cross-sectional survey was administered to caregivers across Saudi Arabia between December 2024 and March 2025. The survey collected demographic characteristics and evaluated caregivers’ KAP regarding household product poisoning. Total KAP scores and domain-specific scores ≥60% were considered satisfactory. Different respondent groups were compared using logistic regression analyses.

**Results:**

1,917 caregivers completed the survey. Although satisfactory attitude (86%) and practice (87%) scores were high, only 37% of respondents demonstrated satisfactory knowledge about childhood poisoning risks. Higher total KAP and knowledge were associated with healthcare-related employment, higher educational attainment, and having ≥3 children. Conversely, lower KAP outcomes were observed among caregivers who were male or aged <25 years. Respondents reported potentially harmful interventions, indicating misconceptions regarding appropriate poisoning management.

**Conclusion:**

Despite positive attitudes and practices, deficiencies remain in caregivers’ understanding of household poisoning risk and appropriate management. A long-term approach for improving child safety that integrates behavior, education, technology, and regulation is needed to reduce household childhood poisoning across Saudi Arabia.

## 1 Introduction

Accidental poisoning from household products is a major global cause of pediatric emergency visits and hospitalizations (1–4). Children’s hand-to-mouth behavior and their natural curiosity can increase pediatric accidental ingestion risk, particularly in unsupervised and insufficiently childproofed settings (5). The American Association of Poison Control Centers reported that 44% of unintentional poisonings occur in children aged <6 years (6). Poisoning incidents can result from exposure to pharmaceutical products or non-pharmaceutical agents. Pharmaceutical products are widely available, easily accessible, and generally presumed to be harmless, leading to underestimation of their potential risk to children (7, 8). Moreover, non-pharmaceutical agents, including various household products such as corrosive cleaning products particularly bleaches and ammonia, laundry detergents, and personal care items, are among the most commonly encountered chemical hazards that can cause significant local and systemic health risks including respiratory, dermal, and gastrointestinal toxicity if ingested, inhaled, or absorbed through the skin (2, 6, 9). In severe cases, corrosive household cleaners can cause burns to the digestive tract, difficulty breathing, and long-term morbidity (10). Household cleaning products are the second leading cause of poisoning in pediatric populations, followed by cosmetics and personal care products (4, 6). The incidence varies globally by socioeconomic indexes, with higher-index countries reporting greater incidence, whereas lower-index countries experience higher childhood morbidity burden and mortality from these events (11). Childhood poisoning remains a leading cause of morbidity and mortality (12), with outcomes shaped by toxic substance type, exposure mode, and the child’s age (13–15). In Saudi Arabia, most poisoning incidents among children aged <5 years are caused by improper handling and storage of organic solvents, disinfectants, and detergents (16–19). International evidence has identified several risk factors contributing to household child poisoning, including poor caregiver attitudes, mislabeled or unlabeled containers, the reuse of detergent bottles for food storage, and inadequate emergency responses (20, 21). Hence, caregivers play a central role in child supervision, the use of child- resistant containers, proper product storage, as well as child education (20). Despite the substantial burden of childhood poisoning globally, caregivers’ ability to identify household chemical hazardous may influence the poisoning risk and prevent such incidents.

Unintentional childhood poisoning persists in Saudi Arabia despite public health initiatives, necessitating community-level interventions. However, the evidence available to inform such interventions remains limited, as most previous studies were regional, retrospective, and lacking direct engagement with caregivers regarding household products poisoning. Evaluating caregivers’ knowledge, attitudes, and practices (KAP) related to household product safety and child poisoning prevention is essential for identifying existing gaps and addressing underlying causes. Therefore, this study evaluated Saudi Arabian caregivers’ KAP to guide focused preventive and educational initiatives. Caregivers were defined in this study as adults aged 18 years or older who directly provided care to at least one child younger than 12 years at home. The caregivers included mothers, fathers, grandparents, siblings, and other adults providing direct care, such as stepmothers.

## 2 Materials and methods

### 2.1 Study design and participants

This cross-sectional study was conducted using an online survey among Saudi residents between December 2024 and March 2025. The survey targeted parents and other family members of students from kindergarten through high school and was distributed via schools using the Ministry of Education’s official research service. Schools were used as a recruitment approach to facilitate the dissemination of the survey link to the parents or caregivers of their students through available electronic communication channels. Recipients completed the online eligibility screening before proceeding with the survey. Including multiple school stages allowed the survey to reach caregivers who may have younger children under 12 years in the household. This approach provided a practical means of reaching the target population of caregivers across different regions of Saudi Arabia. The study received approval from the Biomedical Research Ethics Committee of Umm Al-Qura University (HAPO-02-K-012-2024-10-2321). Participants were also informed that completing the questionnaire was considered as implied consent to take part in the research.

### 2.2 Eligibility criteria

Participants were eligible if they were aged ≥18 years and primary caregivers of children aged <12 years. Eligibility was confirmed through a screening question administered before the main survey, with only completed questionnaires included in the analysis.

### 2.3 Research instrument

In this study, a structured questionnaire was developed based on similar previous studies (22–24). It comprised 26 questions across the following four domains (presented in Supplementary Tables S1–S4): demographics (including age, marital status, educational level, and number of children), knowledge (seven questions assessing household product and medication hazards, corrosive-product identification, symptom recognition, sources of knowledge, poison-control-center awareness, and poisoning management), attitudes (one matrix question comprising nine statements related to child and household safety), and practices (six questions assessing preventive behaviors and household product safety practices).

Five experts reviewed content validity. Face validity was assessed through a pilot study with 10 participants (excluded from the final analysis) to evaluate clarity, response completeness, and survey duration. The questionnaire, originally developed in English, was translated into Arabic and validated through back-translation. Discrepancies were resolved by the same bilingual experts.

### 2.4 Statistical analysis

The minimum required sample size was calculated as 377 using Raosoft software, assuming that 50% of respondents would provide satisfactory responses, with a 95% confidence level and a 10% margin of error (±5%). Categorical data were summarized as frequencies and percentages and compared among study groups using the chi-square test. Every questionnaire item was evaluated independently, and questions with negative wording were reverse-coded. Reliability was evaluated using Cronbach’s alpha, with “alpha-if-item-deleted” analysis employed to identify potential improvements.

Each KAP item was scored according to a predefined key reflecting response accuracy. For hazard-recognition items (household products and medications), respondents rated each item on a 4-point scale: 0 (“I do not know”), 1 (“not harmful at all”), 2 (“low risk”), or 3 (“very harmful”) (Supplementary Table S2). These raw responses were then recoded into an item accuracy score based on their distance from the toxicologically correct hazard level for that item: a response matching the correct level exactly scored 2, a response one scale-point away scored 1, and a response two scale-points away scored 0. “I do not know” responses were also scored 0, since both an incorrect rating and an acknowledged lack of knowledge equally fail to identify the product’s true hazard level and would be equally unlikely to prompt protective caregiving behavior. Corrosive-identification, symptom-recognition, and poisoning-management items were scored 1 for a correct response and 0 otherwise. The poison-control-center awareness item was assigned a maximum of 5 points, disproportionate to its single binary format, to reflect its outsized practical importance as an immediately actionable, potentially life-saving piece of knowledge in a poisoning emergency. The sources-of-information question was reported descriptively and excluded in the knowledge score. Item scores were summed within each sub-domain to provide household-product harm (13 items, maximum 26 points), medication harm (7 items, maximum 14 points), symptom recognition (7 items, maximum 7 points), poisoning management (8 items, maximum 8 points), corrosive-product identification (6 items, maximum 6 points), and poison-control-center awareness (maximum 5 points). These six sub-domain scores were summed to give the knowledge domain raw score, with a maximum of 66 points (26 + 14 + 7 + 8 + 6 + 5). The attitude domain consisted of one matrix question comprising nine statements, each was rated on a 3-point Likert scale (0 = disagree, 1 = indifferent, 2 = agree); negatively phrased statements were reverse-coded prior to summation. Item scores were summed to yield the domain raw scores (attitude: maximum 18 points; practice: maximum 10 points; total KAP: maximum 94 points). An additional question on poisoning history was collected as a background variable and was not scored. For each domain and sub-domain, the percentage score was calculated as (raw score / maximum possible score) × 100; this percentage is the value reported throughout the manuscript and used to define the ≥60% satisfactory-score threshold. Box 1 summarizes this scoring key for every item type.

BOX 1 **Scoring key for knowledge, attitude, and practice items**

**Table:** 

Item type	*N* items	Response scale	Scoring
Household-product harm perception	13	4-point (0 = I do not know, 1 = not harmful, 2 = low risk, 3 = very harmful)	Distance from correct hazard level: 0, 1, or 2 pts./item (max 26)
Medication harm perception	7	Same 4-point scale	Distance-based: 0, 1, or 2 pts./item (max 14)
Symptom recognition	7	Selected/not selected	1 pt. if correct, 0 otherwise (max 7)
Poisoning management	8	Yes / No	1 pt. if correct action, 0 otherwise (max 8)
Corrosive-product identification	6	Selected / not selected	1 pt. if correct, 0 otherwise (max 6)
Poison-control-center awareness	1	Yes / No	5 pts. if yes, 0 if no (max 5)
Attitude statements	9	3-point Likert (0 = disagree, 1 = indifferent, 2 = agree); reverse-coded for negatively phrased items	0–2 pts./item (max 18)
Practice items (scored)	5	Varies by item	0–2 pts./item (max 10)

Subgroup comparisons were conducted using nonparametric tests. Medians and quartiles were used because most domain-score distributions were non-normal. A cutoff of 60% was applied to determine satisfactory scores (25). Binary logistic regression was performed to identify factors associated with satisfactory scores, with participants’ characteristics used as independent variables. Multi-category variables were represented using reference-coded binary indicator variables for each category of substantive interest (e.g., caregiver relationship to the child was represented by separate mother, father, and grandparent indicators, each compared against all other caregiver types combined; age by an 18–25-year indicator compared against caregivers older than 25 years; employment by a housewife-or-employed-outside-the-home indicator). Educational level was entered as a continuous ordinal variable (0 = no formal education to 5 = postgraduate) rather than a binary indicator. Table 1 reports participant characteristics using the same categorization applied in the regression analyses, with reference categories indicated; the full, non-collapsed demographic breakdown is provided in Supplementary Table S24. Regression analyses were conducted twice for each domain: first with all candidate variables entered simultaneously (Supplementary Tables S16–S19), and then using backward likelihood-ratio elimination for variable selection. Log-likelihood values for both the forced-entry and final selected models are reported in the corresponding tables to allow direct comparison of model fit. All analyses were performed using IBM SPSS Statistics for Windows, version 21.0 (IBM Corp., Armonk, N.Y, USA), with supplementary sensitivity analyses (Supplementary Tables S20–S23) conducted in Python (statsmodels 0.14).

**Table 1:** Demographic characteristics of study participants.

Characteristic	Number (%)
Sex	
Male	295 (15.4)
Female (R)	1,622 (84.6)
Age	
18–25 years	123 (6.4)
>25 years (R)	1,794 (93.6)
Employment status	
Housewife or employed outside the home	1,732 (90.3)
Student, retired, or works from home (R)	185 (9.7)
Healthcare-related profession	
Yes	183 (9.5)
No (R)	1,734 (90.5)
Monthly income	
>10,000 SAR	578 (30.2)
≤10,000 SAR (R)	1,330 (69.4)
Relation to child^*^	
Mother	1,454 (75.8)
Father	256 (13.4)
Grandparent	65 (3.4)
Sibling/other	142 (7.4)
Household with child ≤5 years	
Yes	1,138 (59.4)
No (R)	779 (40.6)
Domestic helper	
Yes	853 (44.5)
No (R)	1,064 (55.5)
Number of children	
≥3 children	915 (47.7)
<3 children (R)	1,002 (52.3)
Educational level^†^	
No formal education	11 (0.6)
Primary school	57 (3.0)
Secondary school	72 (3.8)
High school	395 (20.6)
Graduate	1,288 (67.2)
Postgraduate	94 (4.9)

(R) = reference category in the logistic regression models. ^*^Mother, father, and grandparent were each entered as separate binary indicators (that category vs. all other caregiver types combined) rather than as a single reference-coded categorical variable; sibling/other was not entered as a distinct term. ^†^Educational level was entered as a continuous ordinal score (0–5) in the regression models, not as a binary indicator; no reference category applies.

## 3 Results

### 3.1 Questionnaire reliability

Overall Cronbach’s alpha for the entire questionnaire was 0.817, indicating good reliability. Although the “alpha-if-item-deleted” analysis pointed to the possibility of a slight improvement in reliability upon deletion of a few items, the gain in reliability was not considered important compared with the information gained by the full, comprehensive questionnaire.

### 3.2 Characteristics of study participants

Of the 2,769 questionnaire responses, 1,917 were eligible for analysis, representing fully completed questionnaires from eligible participants. Most participants were from the northern region (48.1%), followed by the eastern (18.8%), central (14.8%), western (11.6%), and southern (6.7%) regions of Saudi Arabia (Table 1). Most participants were aged 36–45 years, followed by those aged 26–35 years. Women comprised 84.6% of the sample, and 87.3% of the participants were married. Most respondents were mothers (75.8%), and others included fathers (13.4%), siblings, grandparents, or other caregivers. Approximately two-thirds of the participants held a graduate degree, 20.6% had completed high school, and 4.9% had postgraduate qualifications. Participants who worked outside the home constituted 48.6% of the sample, and homemakers and unemployed participants made up 41.7%. Only 9.5% of participants had healthcare-related professions. Regarding monthly income as a marker of social standard, 30.3% of participants had no income, 22.5% earned 5,000–10,000 Saudi Riyal (SAR), and 19.8% earned 11,000–15,000 SAR. The number of children aged <12 years per family varied, with 29.5 and 22.8% having two and three children, respectively. Participants cared for children of all age groups, with the highest proportions in the 10–12-year-old (52.9%) and 8–9-year-old (44.8%) age groups. Over 80% of participants had one or more children aged ≤5 years. More than half of respondents (55.5%) reported having no domestic helper, and 32.6% had one.

### 3.3 Knowledge of household products and medication poisoning risks, symptoms, and management in children

Adequate knowledge regarding the risks of household products was reported. Bleach and insecticides were identified as highly harmful, and shampoo and toothpaste were perceived as low risk (Table 2). Oral contraceptives and pain relievers were commonly recognized as hazardous household medications. The risk posed by iron supplements was typically underestimated, indicating a lack of awareness about their potential toxicity in children. Although several products, including Clorox and Flash, were identified as corrosive poisons, only a few respondents were aware of sodium hydroxide and its associated risks.

**Table 2:** Knowledge of poisoning risks, symptoms, and management related to household products and medications.

Item	Frequency (%)			
	Not harmful at all	Low risk^†^	Very harmful^‡^	I do not know
Respondents’ recognition of harm from exploratory ingestion of household products				
Bleach	51 (2.7)	168 (8.8)	1,650 (86.1)	48 (2.5)
Cleaning products (e.g., toilet cleaner)	47 (2.5)	200 (10.4)	1,612 (84.1)	58 (3.0)
Insecticides	48 (2.5)	145 (7.6)	1,673 (87.3)	51 (2.7)
Soap	209 (10.9)	881 (46.0)	782 (40.8)	45 (2.3)
Nail polish	142 (7.4)	591 (30.8)	1,084 (56.5)	100 (5.2)
Shampoo	280 (14.6)	918 (47.9)	656 (34.2)	63 (3.3)
Mothballs (Naphthalene)	49 (2.6)	204 (10.6)	1,507 (78.6)	157 (8.2)
Toothpaste	667 (34.8)	933 (48.7)	243 (12.7)	74 (3.9)
Play dough (Play-Doh)	245 (12.8)	738 (38.5)	803 (41.9)	131 (6.8)
Mouthwash	566 (29.5)	888 (46.3)	357 (18.6)	106 (5.5)
Glue	123 (6.4)	539 (28.1)	1,149 (59.9)	106 (5.5)
Button batteries	59 (3.1)	182 (9.5)	1,587 (82.8)	89 (4.6)
Lipstick	727 (37.9)	798 (41.6)	273 (14.2)	119 (6.2)
Respondents’ recognition of harm from exploratory ingestion of medications				
Vitamins	487 (25.4)	943 (49.2)	337 (17.6)	150 (7.8)
Iron supplement tablets	321 (16.7)	868 (45.3)	526 (27.4)	202 (10.5)
Pain relievers (e.g., Panadol or Tylenol)	173 (9.0)	834 (43.5)	784 (40.9)	126 (6.6)
Oral contraceptive pills	87 (4.5)	455 (23.7)	1,158(60.4)	217 (11.3)
Antibiotic ointment	208 (10.9)	647 (33.8)	882 (46.0)	180 (9.4)
Cough and cold medications	360 (18.8)	964 (50.3)	490 (25.6)	103 (5.4)
Antihistamines (e.g., Zyrtec or Telfast)	170 (8.9)	657 (34.3)	876 (45.7)	214 (11.2)

	Frequency (%)
Respondents’ identification of household products with corrosive effects^*^	
Vinegar (Acetic acid)	194 (10.1)
Flash (Sodium carbonate)	1,757 (91.7)
Clorox (Sodium hypochlorite)	1,215 (63.4)
Harpic (Hydrochloric acid)	864 (45.1)
Caustic soda (Sodium hydroxide)	8 (0.4)
I do not know	891 (46.5)
Respondents’ recognition of symptoms associated with household cleaning product ingestion^*^	
Vomiting	1,541 (80.4)
Dizziness	1,029 (53.7)
Difficulty breathing	1,541 (80.4)
Seizures	346 (18.0)
Burning around the mouth and face	1,171 (61.1)
Abdominal pain	1,458 (76.1)
Difficulty swallowing	894 (46.6)
Respondents’ sources of knowledge regarding household product toxicity^*^	
Asking healthcare professionals (e.g., physician or pharmacist)	1,360 (70.9)
Asking a sales representative	94 (4.9)
Social media	484 (25.2)
School/university	223 (11.6)
Search engine (e.g., Google)	1,134 (59.2)
Educational programs	484 (25.2)
Asking a friend/relative	479 (25.0)
Others (e.g., scientific papers)	10 (0.7)
Respondents’ knowledge of the poison consultation service contact number	
Yes	788 (41.1)

	Frequency (%)		
	Yes	No	I do not know
Respondents’ views on the management of cleaning product ingestions			
Drinking milk	1,279 (66.7)	270 (14.1)	368 (19.2)
Drinking saltwater	373 (19.5)	708 (36.9)	836 (43.6)
Drinking lemon juice	310 (16.2)	788 (41.1)	819 (42.7)
Drinking vinegar	80 (4.2)	1,026 (53.5)	811 (42.3)
Drinking large amounts of water	1,145 (59.7)	314 (16.4)	458 (23.9)
Using activated charcoal	275 (14.3)	705 (36.8)	937 (48.9)
Using over-the-counter medications	294 (15.3)	897 (46.8)	726 (37.9)
Using herbal remedies	310 (16.2)	839 (43.8)	768 (40.1)

^†^Low risk (harmful only in large quantities).

^‡^Very harmful (harmful even in small quantities).

^*^Multiple responses were allowed.

The symptoms of corrosive poisoning, including respiratory difficulties, vomiting, and abdominal pain, were widely recognized, whereas seizures and swallowing difficulties were less known. Only 41.1% of respondents knew the poison control center’s contact number. Misconceptions about managing corrosive poisoning involved recommending drinking large quantities of water or milk to reduce severity. Although inappropriate in these situations, some respondents suggested the use of activated charcoal as a detoxification strategy.

### 3.4 Attitude toward child and household product safety

Participants demonstrated positive attitudes toward child safety (Table 3). Approximately two-thirds of respondents agreed that all household medications pose a potential risk to children, and 89.3% agreed that safe storage was a shared household responsibility. Attitudes toward child-resistant packaging were ambivalent, with many respondents doubting its effectiveness. Unsafe storage practices were common, with 21.1 and 32.8% of respondents endorsing the storage of cleaning products under the sink and in visible locations, respectively. Most respondents emphasized that poisoning occurs due to improper product storage and that educating children is mainly the responsibility of caregivers. Although 64.3% of respondents considered safety information on product labels to be clear, approximately half relied on social media as a primary information source, which raises concerns.

**Table 3:** Attitude toward child and household products safety.

Questionnaire item	Frequency (%)		
	Agree	Neutral	Disagree
Medications used at home can be harmful to children, regardless of their type	1,238 (64.6)	487 (25.4)	192 (10.0)
Children find it difficult to open medication packaging	750 (39.1)	499 (26.0)	668 (34.8)
The responsibility for keeping medications out of children’s reach lies with everyone in the household	1,711 (89.3)	113 (5.9)	93 (4.9)
The optimal place to store household cleaners is under the kitchen sink	404 (21.1)	346 (18.0)	1,167 (60.9)
Cleaners can be placed in visible locations as long as they are out of children’s reach	629 (32.8)	356 (18.6)	932 (48.6)
Household products such as cleaners and cosmetics may pose a risk for children if not stored properly	1,559 (81.3)	171 (8.9)	187 (9.8)
The safety information on household products is clear and easy to understand	1,232 (64.3)	447 (23.3)	238 (12.4)
Social media is an important source of information	966 (50.4)	626 (32.7)	325 (17.0)
The responsibility for educating children about the dangers of toxic household products lies with parents and caregivers	1,609 (83.9)	139 (7.3)	169 (8.8)

### 3.5 Practices related to the safe use and storage of household products

Most caregivers reported safe practices to reduce childhood poisoning risk in the home (Table 4). The majority reported using locked or raised storage (86.4%), reading safety warnings (51.3%), avoiding the reuse of cleaning product containers (78.1%), and storing items immediately after use (82.4%). Of those surveyed, approximately half (49.3%) reported educating their children on the risks of poisoning. Nonetheless, 14.3% of respondents reported at least one poisoning incident, highlighting that lapses in practice, even if uncommon, can lead to preventable injury.

**Table 4:** Practices related to the safe use and storage of household products.

Item	Response options	Frequency (%)
Use of cleaning product containers for other purposes	Always	55 (2.9)
	Sometimes	305 (15.9)
	Never	1,498 (78.1)
	I do not remember	59 (3.1)
Frequency of reading safety instructions for cleaning products	Every time I use a new product	984 (51.3)
	When I think it might be dangerous	475 (24.8)
	I rarely read them	270 (14.1)
	Never	57 (3.0)
	I do not remember	131 (6.8)
Current storage location of household cleaners*	Under the kitchen sink (closed cabinet with a lock)	630 (32.9)
	Under the kitchen sink (open cabinet or without a lock)	336 (17.5)
	In the bathroom cabinet (closed with a lock)	552 (28.8)
	In the bathroom cabinet (open or without a lock)	189 (9.9)
	On a high shelf	843 (44.0)
	In a closed storage box	539 (28.1)
	On the kitchen or bathroom floor	91 (4.7)
Storage summary	Locked (any location)	1,231 (64.2)
	High shelf (regardless of lock)	426 (22.2)
	Neither locked nor high	260 (13.6)
Frequency of putting products away immediately after use	Always	1,579 (82.4)
	Sometimes	284 (14.8)
	Never	25 (1.3)
	I do not remember	29 (1.5)
Frequency of teaching children about the dangers of household products in the past year	Always	945 (49.3)
	Sometimes	588 (30.7)
	Never	117 (6.1)
	I do not remember	267 (13.9)
Reported incidents of childhood poisoning in the home due to cleaning products and medications	Yes, it happened once	173 (14.3)
	Yes, it happened multiple times	22 (1.1)
	No, no poisoning has occurred	1,622 (84.6)
	I do not remember	100 (5.2)

^*^Multiple responses allowed.

### 3.6 Knowledge, attitudes, and practice (KAP) scores and sex differences among participants

Caregiver preparedness varied across domains (Figure 1a). Practice had the highest average score (80%), followed by attitude (78%), and knowledge (56%). Within the knowledge domain, awareness was the strongest for household products, lower for medications and corrosives, and the lowest for poisoning management (37.5%). Women scored significantly higher than men in total KAP, knowledge, and all subdomains (*p* < 0.01) (Table 5). Supplementary Tables S5–S15 present the associations between KAP scores and participant subgroups.

**Figure 1:**
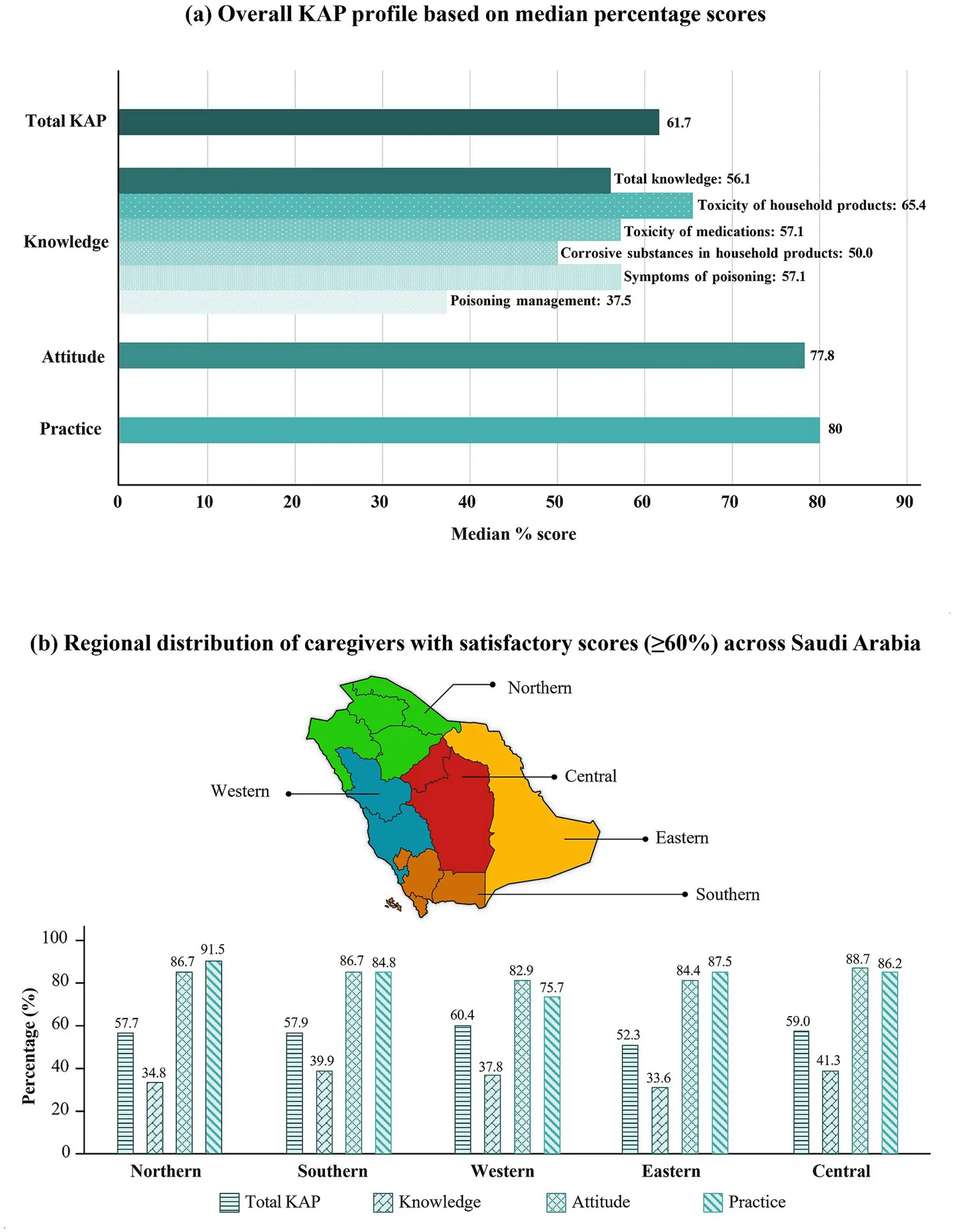
Caregiver preparedness regarding household poisoning in children in Saudi Arabia. **(a)** Median percentage scores for knowledge, attitude, practice, and overall KAP among caregivers. **(b)** Regional distribution of caregivers with satisfactory scores (≥60%) across Saudi Arabian regions.

**Table 5:** Median score of knowledge, attitude, and practice (KAP) among all study participants, with comparison of female and male scores.

Domain	Median % of maximum (quartiles)		*p-*value
	Women (*n* = 1,622)	Men (*n* = 295)	
Total KAP score	62.8 (55.3, 69.1)	59.8 (52.1, 67.0)	<0.001^*^
Total knowledge	56.8 (48.5, 63.6)	53.1 (42.4, 62.1)	<0.001^*^
Toxicity of household products	65.9 (53.8, 76.9)	61.5 (50.0, 73.1)	<0.001^*^
Toxicity of medications	57.1 (42.9, 64.3)	50.0 (35.7, 57.1)	0.007^*^
Corrosive substances in household products	50.0 (50.0, 66.7)	50.0 (50.0, 66.7)	0.009^*^
Symptoms of poisoning	71.4 (42.9, 85.7)	57.1 (28.6, 71.4)	<0.001^*^
Poisoning management	37.5 (0, 62.5)	25.0 (0, 62.5)	0.002^*^
Attitude	77.8 (66.7, 88.9)	77.8 (66.7, 88.9)	0.773
Practice	80.0 (70.0, 90.0)	80.0 (60.0, 90.0)	0.171

^*^Statistically significant at *p* < 0.05.

Values represent the median (interquartile range), rather than the mean, of the percentage score for each domain, calculated as (raw domain score / maximum possible domain score) × 100 for each participant.

### 3.7 Comparison of the percentage of participants with satisfactory scores (≥60%) in total KAP, knowledge, attitude, and practice across different groups

A satisfactory score in knowledge (≥60%) was obtained by 37.0% of respondents compared with 86.4, 87.4, and 57.9% for attitude, practice, and total KAP, respectively (Table 6). Moreover, regional variations in satisfactory scores were observed across Saudi Arabia (Figure 1b). Participants who were female, had higher education, were healthcare professionals, were mothers or grandparents, and were caregivers with ≥3 children had significantly higher satisfactory scores in total KAP (Table 6). Knowledge scores were associated with being female, having a higher educational level, being a healthcare professional, having a higher income, having at least one domestic helper, and having more children (*p* < 0.05). Mothers and grandparents had higher attitude scores, whereas respondents aged <25 years had lower scores (*p* < 0.05). Practice scores were lower for respondents aged <25 years but were highly associated with female sex, married caregivers, individuals working from home, and households with at least one domestic helper (*p* < 0.05).

**Table 6:** Percentage of participants with satisfactory scores (60% or higher) in total KAP, knowledge, attitude, and practice, across different subgroups of participants.

Group	Total KAP		Knowledge		Attitude		Practice	
	Satisfactory [*n* (%)]	*p*-value	Satisfactory [*n* (%)]	*p*-value	Satisfactory [*n* (%)]	*p*-value	Satisfactory [*n* (%)]	*p*-value
Overall	1,110 (57.9)		709 (37.0)		1,656 (86.4)		1,675 (87.4)	
Age (years)								
18–25	59 (48.0)	0.054	41 (33.3)	0.486	96 (78.0)	0.001^*^	94 (76.4)	0.002^*^
26–35	302 (56.8)		208 (39.1)		447 (84.0)		478 (89.8)	
36–45	504 (61.0)		297 (36.0)		730 (88.4)		728 (88.1)	
46–55	213 (55.8)		139 (36.4)		341 (89.3)		329 (86.1)	
≥56	32 (59.3)		24 (44.4)		42 (77.8)		46 (85.2)	
Sex								
Female	968 (59.7)	<0.001^*^	618 (38.1)	0.018^*^	1,407 (86.7)	0.281	1,430 (88.2)	0.015*
Male	142 (48.1)		91 (30.8)		249 (84.4)		245 (83.1)	
Marital status								
Single	70 (57.4)	0.943	44 (36.1)	0.965	99 (81.1)	0.219	89 (73.0)	<0.001^*^
Single parent	69 (56.6)		46 (37.7)		106 (86.9)		110 (90.2)	
Married	971 (58.0)		619 (37.0)		1,451 (86.7)		1,476 (88.2)	
Highest level of education								
No formal education	2 (18.2)	<0.001^*^	1 (9.1)	<0.001^*^	8 (72.7)	0.305	8 (72.7)	0.543
Primary school	26 (45.6)		14 (24.6)		45 (78.9)		47 (82.5)	
Secondary school	30 (41.7)		15 (20.8)		60 (83.3)		62 (86.1)	
High school	213 (53.9)		136 (34.4)		345 (87.3)		350 (88.6)	
Graduate	773 (60.0)		496 (38.5)		1,119 (86.9)		1,126 (87.4)	
Postgraduate	66 (70.2)		47 (50.0)		79 (84.0)		82 (87.2)	
Employment status								
Work outside the home	543 (58.8)	0.732	357 (38.7)	0.666	790 (85.6)	0.115	800 (86.7)	<0.001^*^
Work from home	19 (52.8)		14 (38.9)		31 (86.1)		33 (91.7)	
Unemployed/Homemaker	459 (57.4)		281 (35.1)		707 (88.4)		721 (90.1)	
Student	39 (55.7)		26 (37.1)		54 (77.1)		48 (68.6)	
Teacher/doctor	7 (77.8)		2 (22.2)		8 (88.9)		8 (88.9)	
Retired	43 (54.4)		29 (36.7)		66 (83.5)		65 (82.3)	
Healthcare-related profession								
No	979 (56.5)	<0.001^*^	612 (35.3)	<0.001^*^	1,501 (86.6)	0.485	1,523 (87.8)	0.065
Yes	131 (71.6)		97 (53.0)		155 (84.7)		152 (83.1)	
Monthly income								
<5,000	185 (56.6)	0.111	115 (35.2)	0.009^*^	286 (87.5)	0.086	284 (86.9)	0.358
5,000–10,000	245 (56.7)		176 (40.7)		362 (83.8)		379 (87.7)	
11,000–15,000	240 (63.3)		149 (39.3)		321 (84.7)		324 (85.5)	
>15,000	120 (60.3)		84 (42.2)		181 (91.0)		170 (85.4)	
No income	314 (55.0)		180 (31.5)		501 (87.7)		511 (89.5)	
Relation to children								
Mother	863 (59.4)	0.008^*^	550 (37.8)	0.197	1,269 (87.3)	0.049^*^	1,305 (89.8)	<0.001^*^
Father	124 (48.4)		81 (31.6)		213 (83.2)		211 (82.4)	
Grandparent	44 (67.7)		29 (44.6)		57 (87.7)		52 (80.0)	
Sibling	42 (53.8)		25 (32.1)		60 (76.9)		55 (70.5)	
Other	36 (57.1)		23 (36.5)		56 (88.9)		51 (81.0)	
Number of children								
1	239 (54.8)	0.01^*^	144 (33.0)	0.001^*^	382 (87.6)	0.711	374 (85.8)	0.396
2	320 (56.5)		206 (36.4)		482 (85.2)		491 (86.7)	
3	285 (65.1)		199 (45.4)		376 (85.8)		389 (88.8)	
4	156 (57.8)		93 (34.4)		238 (88.1)		243 (90.0)	
5–10	110 (53.1)		67 (32.4)		178 (86.0)		178 (86.0)	
Households with at least one domestic helper								
No	599 (56.3)	0.112^*^	364 (34.2)	0.005^*^	922 (86.7)	0.701	949 (89.2)	<0.008^*^
Yes	511 (59.9)		345 (40.4)		734 (86.0)		726 (85.1)	

^*^Statistically significant at *p* < 0.05; KAP, knowledge, attitude, and practice.

### 3.8 Predictors of KAP scores

Forced-entry regression (Supplementary Tables S16–S19) and backward likelihood-ratio elimination models (Table 7) were used for multivariable regression analysis. For total KAP, male caregivers had 39.3% lower odds of a satisfactory score than female caregivers (OR = 0.607, 95% CI 0.471–0.783), healthcare-related employment was associated with double the odds of a satisfactory score (OR = 2.000, 95% CI 1.400–2.858), age <25 years was associated with 38.4% lower odds (OR = 0.616, 95% CI 0.419–0.904), having ≥3 children with 24.9% higher odds (OR = 1.249, 95% CI 1.037–1.505), and each unit increase in educational level with 27.1% higher odds (OR = 1.271, 95% CI 1.130–1.429). The knowledge domain showed a similar pattern at somewhat smaller magnitude: 28.3% lower odds for male sex (OR = 0.717, 95% CI 0.547–0.940), 92.6% higher odds for healthcare-related employment (OR = 1.926, 95% CI 1.403–2.645), 25.1% higher odds for ≥3 children (OR = 1.251, 95% CI 1.035–1.511), and 28.8% higher odds per unit of education (OR = 1.288, 95% CI 1.133–1.464). For attitude, age <25 years remained the only variable retained at conventional statistical significance, with 50.9% lower odds of a satisfactory score (OR = 0.491, 95% CI 0.310–0.780, *p* = 0.003); employment status, higher educational level, being a father, and male sex were also retained by the elimination procedure but approached significance without reaching it (*p* = 0.05–0.11), and are reported as exploratory associations given the comparatively low variance explained by the attitude model. For practice, being a mother was associated with more than double the odds of a satisfactory score (OR = 2.045, 95% CI 1.522–2.748), while age <25 years (OR = 0.576, 95% CI 0.363–0.912) and having at least one domestic helper (OR = 0.719, 95% CI 0.547–0.946) were associated with 42.4 and 28.1% lower odds, respectively. A sensitivity analysis using continuous rather than dichotomized domain scores (Supplementary Tables S20–S23) confirmed no material change in the direction of predictors retained in Table 7, with one exception: male sex did not reach significance in the continuous-outcome models for total KAP and knowledge despite remaining significant in the dichotomized analysis.

**Table 7:** Logistic regression analysis (backward likelihood-ratio elimination) for satisfactory scores across various demographic variables.

Variable	OR	95% CI		*p*-value
		Lower	Upper	
Satisfactory score in total KAP				
Sex (male)	0.607	0.471	0.783	<0.001^*^
Healthcare-related profession	2.000	1.400	2.858	<0.001^*^
Grandparent	1.572	0.910	2.715	0.105
Age <25 years	0.616	0.419	0.904	0.013^*^
Households with ≥3 children	1.249	1.037	1.505	0.019^*^
Higher educational level	1.271	1.130	1.429	<0.001^*^
Log-likelihood: forced-entry model = −1265.65 (df = 13); final selected model = −1266.93 (df = 7)				
Satisfactory score in knowledge				
Sex (male)	0.717	0.547	0.940	0.016^*^
Healthcare-related profession	1.926	1.403	2.645	<0.001^*^
Households with ≥3 children	1.251	1.035	1.511	0.020^*^
Higher educational level	1.288	1.133	1.464	<0.001^*^
Log-likelihood: forced-entry model = −1229.28 (df = 13); final selected model = −1232.40 (df = 5)				
Satisfactory score in attitude				
Sex (male)	2.192	0.834	5.757	0.111
Employment status	0.928	0.862	1.000	0.050
Being a father	0.384	0.143	1.036	0.059
Age <25 years	0.491	0.310	0.780	0.003^*^
Higher educational level	1.161	0.984	1.371	0.077
Log-likelihood: forced-entry model = −744.71 (df = 13); final selected model = −744.99 (df = 6)				
Satisfactory score in practice				
Mother	2.045	1.522	2.748	<0.001^*^
Age <25 years	0.576	0.363	0.912	0.019^*^
Household with domestic helper	0.719	0.547	0.946	0.018^*^
Log-likelihood: forced-entry model = −698.77 (df = 13); final selected model = −701.87 (df = 4)				

^*^Statistically significant at *p* < 0.05. CI, confidence interval; OR, odds ratio; KAP, knowledge, attitude, and practice.

## 4 Discussion

Unintentional childhood poisoning is a global public health issue, influenced by cultural, social, and healthcare determinants. Over half of accidental poisoning cases in Saudi Arabia involved children aged <5 years, particularly from detergent ingestion, with seasonal variations (16). Exposure to disinfecting agents and organic solvents commonly affected children aged <5 years (17). Research from Riyadh identified behavioral hazards, including container misuse and label disregards (18), and a recent study in Sakakah, Al-Jouf, highlighted key predictors among preschoolers, including male sex, large household, and unsafe storage of powdered medications (19). However, these regionally restricted studies did not assess caregivers’ knowledge, attitudes, or practices toward household product poisoning. The present study provides an assessment of caregivers’ KAP with a large sample, providing robust, generalizable insights across Saudi Arabia using a validated, multidimensional questionnaire.

Most respondents were from the northern region, from which 40% of acute chemical exposures in children are reported, a substantially higher proportion than in other regions (17). Although the exact causes remain undetermined, factors including regional variation among caregivers’ knowledge, family size, and socioeconomic status may contribute. Incidence was not directly measured; however, the geographic overlap highlights the need for targeted awareness and preventive initiatives in high-exposure areas.

Most respondents were mothers (75.8%), reflecting their natural role in household child safety (26). Although two-thirds held graduate degrees, the influence of educational attainment influence on poisoning prevention varies across studies (27, 28). Participants differed in socioeconomic status, potentially impacting their emergency care access and safety practices. Most households had children aged <5 years and lacked domestic helpers, indicating that primary caregivers were fully responsible for child safety.

Knowledge among parents and guardians about accidental household childhood poisoning risks is critical for prevention. Consistent with studies from Lebanon (29) and Uganda (30), most Saudi respondents identified bleach, cleaners, insecticides, mothballs, and button batteries as high-risk products. Despite certain mouthwashes containing 27% alcohol (31), capable of causing severe intoxication even with minimal exposure (32), 78% of respondents underestimated their associated risk. Products including hand soap, playdough, glue, and nail polish, were perceived as highly toxic despite their low risks, suggesting that unclear labeling or chemical terminology can lead to overestimation and misguided preventive measures.

Awareness of medication risks was good; however, 62% of respondents underestimated the hazard of iron supplements, a major source of morbidity in young children, despite declining severe exposures (33). Most respondents believed that over-the-counter drugs are only harmful in high doses, although even small amounts can be dangerous. Such exposures are reported in poison control data, particularly in <6-year-old children (34).

Knowledge of household cleaning product chemical names was replaced by brand recognition. Nearly all respondents linked Flash and Clorox with corrosive poisoning; only 0.4% identified sodium hydroxide. Although participants recognized corrosive poisoning symptoms, misunderstandings about treatment persisted: 59.7 and 66.7% endorsed consuming large quantities of water or milk, respectively, to treat such poisoning. These practices can cause vomiting, reexposure, and additional injury and are not recommended (35, 36). Activated charcoal was suggested by 14.3% of respondents, although it is ineffective for small, highly ionized corrosives and can interfere with endoscopy (36, 37). Various home therapies such as saltwater, vinegar, and lemon juice are also contraindicated due to exothermic neutralizing reactions that can worsen mucosal injury (35). This knowledge gap shows a significant disconnect between public awareness and recommended practices, potentially exacerbating toxicity and delaying evidence-based treatment. Only 41.1% were aware of poison control contacts, limiting timely expert consultation for optimal outcomes.

Caregivers had positive attitudes toward child and household product safety. Nearly 89% of respondents agreed that keeping medications out of children’s reach is a shared duty, and 81% acknowledged the dangers of improperly storing cleaning products and cosmetics. Only 39.1% believed child-resistant packaging is difficult for children to open, aligning with evidence that such designs typically fail to prevent children from accessing the content (38). One-third of the respondents preferred storing products in visible locations or under the sink, prioritizing convenience over safety. Most respondents relied on social media for information on product safety and poisoning prevention measures; however, its lack of clarity raised concerns about potential misinformation.

Most caregivers reported beneficial safety practices regarding storing items immediately after use, reading safety instructions, using locked storage, and avoiding the reuse of containers for other purposes. These behaviors positively reduce childhood poisoning risk, as previously reported (39). Unsafe practices persist globally. In Uganda, 67.8% of caregivers demonstrated poor chemical storage practices (30). Australian and United Kingdom studies show that several risk factors, including unsafe product storage and absence of original packaging, impacted childhood poisoning risk (39, 40). In our cohort, 14.3% of respondents reported at least one home poisoning incidence, illustrating the impact of rare, dangerous practices.

The average knowledge score was lower than the practice (80%) and attitude (78%) scores, particularly for perceived poisoning management knowledge (37.5%). The lower scores of knowledge are demonstrated by the specific information assessed in this domain, in particular, the identification of the risk of household products, medications and corrosive substances and the appropriate management action required following corrosive exposure. On the other hand, the attitude and practice domains mainly assessed risk perception and safety beliefs, which may be acquired without requiring specific knowledge about toxic products. Thus, positive attitudes and prudent practices can be the result of general parental instinct and common safety norms, while the correct identification of specific hazardous products, corrosive agents, and appropriate actions of poisoning management requires focused health education, which may not be widespread in the community.

Furthermore, high practice scores may be at least partly due to habitual household organization rather than risk-based decision-making; practices that are not based on accurate knowledge may be less effective in less routine situations such as exposure to unfamiliar products.

It is possible that self-reported attitudes and practices were vulnerable to social desirability bias, as caregivers may be prone to select responses that are perceived to reflect responsible caregiving. Conversely, knowledge items are objectively right or wrong and therefore less vulnerable to social desirability bias, which may better reflect true gaps in caregiver understanding. The phenomenon of a stronger attitude than knowledge is not consistent internationally. Kurup et al. (41) found relatively consistent KAP scores with fewer severe deficiencies, whereas Shehata et al. (22) reported high attitudes (97.5%) but lower knowledge (51%) and poor practices (36.3%). Consistent with our findings, studies from Australia and Pakistan identified improper storage practices and underestimation of over-the-counter drug risks (42, 43).

Using a 60% cutoff, 57.9% of respondents achieved a satisfactory total KAP score, with knowledge being the lowest domain (37.0%) compared with attitude (86.4%) and practice (87.4%). Similar to findings from Pakistan, these positive behaviors may reflect routine or cultural norms rather than comprehensive awareness of poisoning hazards (43). Although no regional variation was observed in total KAP, knowledge, and attitude scores, practice scores were lower in the southern and western regions, aligning with previous findings (17). Women outperformed men in total KAP and knowledge scores, but not in attitude or practice, suggesting comparable safety behaviors despite lower hazard awareness among men. This aligns with evidence that women, frequently serving as primary caregivers, are more health-literate and engaged with child safety information (44). Given that women constituted the majority of the study sample (85%), this brings in the question of whether the gender imbalance affected the overall study findings. Respondents aged <25 years scored low in attitude and practice, likely reflecting less caregiving experience and lower awareness of associated risks. Higher educational attainment and healthcare-related employment were highly correlated with increased total KAP and knowledge scores, consistent with earlier studies linking formal education to better hazard recognition (42, 45). Mothers and grandparents achieved higher attitude and practice scores, and large family size was associated with greater knowledge score, suggesting the benefits of experience-based learning, consistent with findings from Qatar (46).

Multivariable analysis confirmed these trends. Healthcare-related profession, higher educational level, and having ≥3 children were associated with higher odds of satisfactory KAP. Conversely, male sex and age <25 years were inverse predictors. The persistence of this association after multivariable adjustment suggests that the female knowledge advantage was not solely attributable to women being overrepresented in the sample and may partly reflect greater exposure to caregiving-related health information.

The attitude-domain models explained comparatively little variance overall. Age <25 years remained a statistically significant inverse predictor (OR = 0.491, *p* = 0.003), while employment status, being a father, higher education, and male sex approached but did not reach conventional significance. This pattern suggest that attitudes toward household product safety are more evenly distributed across the sociodemographic and occupational subgroups examined, in contrast to the more clearly patterned predictors identified for knowledge and practice. This may instead reflect the influence of factors not captured in this model, such as prior personal experience with a poisoning incident. Notably, the direction of the sex effect on attitude (male caregivers trending toward higher, rather than lower, scores) was opposite to its consistently negative association with knowledge and practice, although this trend did not reach significance and should be interpreted cautiously. The caregiver role influenced practice. Being a mother was associated with approximately twice the odds of satisfactory practice, whereas having a domestic helper reduced the odds, reflecting that households with domestic helpers were less directly involved in household safety measures.

This study has several strengths, including a large, demographically diverse sample from across Saudi regions. The use of a validated KAP tool facilitated comparative assessments. The results highlight gaps in caregivers’ understanding of safe storage procedures and evidence-based poisoning management. Parental KAP toward child safety should be improved by structured health education focusing on sex-sensitive and culturally appropriate interventions (47). Approaches involving behavior design to promote safer habits, technology-based tools that offer instant guidance, and risk assessment tools integrated into routine pediatric healthcare practices should be implemented to improve child safety.

This study had some limitations. First, the reliance on self-reported data may have introduced recall or social desirability biases, potentially overestimating positive activities. Second, the school-based recruitment approach limits the reach of caregivers who may have pre-kindergarten children, which may influence the generalizability of the findings to caregivers of very young children. Third, the online study design may have excluded individuals with lower socioeconomic status or limited internet access, potentially compromising the representativeness of the findings. Furthermore, the relatively low number of male participants in this study may affect the accuracy of estimates of men in subgroups. Future research could consider observational data, indirect questioning, or mixed-method approaches and use complementary recruitment through pediatric healthcare settings, nurseries, or daycare centers to better represent the young children subgroup.

## 5 Conclusion

This study identified crucial gaps in caregivers’ knowledge of household product-related poisoning in children. Despite promising safety attitudes and practices, substantial gaps persist in poisoning-related knowledge, particularly regarding thehazardous of household substances and appropriate response following poisoning exposure. Unintentional household poisoning in children could be reduced through targeted educational initiatives, particularly for younger caregivers and potentially for male caregivers. Improving child safety requires a sustainable strategy combining behavioral, educational, technological, and policy approaches. Future public health interventions should incorporate targeted caregiver education through schools, healthcare settings, and other community-based settings to strengthen child safety across diverse populations.

## Data Availability

The raw data supporting the conclusions of this article will be made available by the authors, without undue reservation.
