# Supplementary material for: Caregivers’ knowledge, attitudes, and practices regarding household product safety and poisoning prevention in children in Saudi Arabia
Source: Front Public Health. 2026 Oct 19;14:1937239. doi: 10.3389/fpubh.2026.1937239 (PMC13724360; doi:10.3389/fpubh.2026.1937239)
Supplement: Supplementary file 1 [file Supplementary_file_1.docx]

Supplementary Material

# 1 Supplementary Tables

**Questionnaire**

Table S1: Demographic information

| Region | Eastern region  Western region  Southern region  Northen region  Central region |
| --- | --- |
| Age | 18-25 years  26-35 years  36-45 years  46-55 years  56 years and above |
| Gender | Male  Female |
| Marital status | Single  Single-parent family (separated or widowed)  Married |
| What is the highest level of education attained? | No formal education  Primary school  Secondary school  High school  Graduate (diploma, bachelor)  Post-graduate (master, doctorate, other) |
| Employment status | Employed (work outside the home)  Employed (work from home)  Unemployed / Housewife  Student  Retired  Other (please specify): ________ |
| Is your profession related to healthcare (e.g., nurse, dietitian, doctor, pharmacist, etc.) | Yes  No |
| Monthly income | Less than 5,000 SAR  5,000 – 10,000 SAR  11,000 – 15,000 SAR  More than 15,000 SAR  I have no income |
| What is your relationship to the children you care for at home? | Mother  Father  Grandparent  Siblings  Other caregivers (please specify): ________ |
| Number of children (< 12 years) you are caring for? | 1  2  3  4  5 -10 |
| Age of children (select all that apply): | 1 year or below  2-3 years  4-5 years  6-7 years  8-9 years  10-12 years |
| Number of housemaids | 0  1  2  >3 |

Table S2: Knowledge about poisoning risks from household products and medications

|  | | Scale |
| --- | --- | --- |
| Based on your knowledge, what is the potential level of harm if children exploratively ingest the following household products? | Bleach  Cleaning products (such as toilet cleaner, laundry detergent, degreaser)  Insecticides  Soap  Nail polish  Shampoo  Mothballs (Naphthalene)  Toothpaste  Play dough (Play-Doh)  Mouthwash  Glue  Button batteries  Lipstick | (1) Not harmful at all  (2) Low risk (harmful only in large quantities)  (3) Very harmful (harmful even in small quantities)  (0) I don't know |
| Based on your knowledge, what is the potential level of harm if children exploratively ingest the following household medications? | Vitamins  Iron supplement tablets  Pain relievers (such as Panadol, Tylenol, Profenac, Aspirin)  Oral contraceptive pills  Antibiotic ointment  Cough and cold medications  Antihistamines (such as Zyrtec, Telfast) | (1) Not harmful at all  (2) Low risk (harmful only in large quantities)  (3) Very harmful (harmful even in small quantities)  (0) I don't know |
| Which of the following household products has corrosive effects that can cause chemical burns and tissue damage? (select all that apply) | Vinegar  Flash  Clorox  Harpic  Soda (Sodium hydroxide)  I don't know |  |
| Select from the following symptoms what you think may indicate a potential poisoning after ingesting household cleaning products. (Select all that apply) | Vomiting  Dizziness  Difficulty breathing  Seizures  Burning around the mouth and face  Abdominal pain  Difficulty swallowing  None of them  I don't know |  |
| Do you know the contact number for the poisoning consultation service? | Yes  I don't know |  |
| What sources do you rely on for information about the risks and toxicity of household products? (Select all that apply) | Questioning healthcare professionals (such as doctors and pharmacists)  Questioning a sales representative  Social media  School / University  Search engine (such as google)  Educational programs  Asking a friend / relative  Other (please specify) __________ |  |
| Based on your knowledge, do you think that each of the following methods can be used to alleviate cases of poisoning from household cleaning products in children? | Drinking milk  Drinking saltwater  Drinking lemon juice  Drinking vinegar  Drinking large amounts of water  Using activated charcoal  Using over-the-counter medications  Using herbal remedies | Yes  No  I don't know |

Table S3: Attitude towards child and household products safety

|  | | Scale |
| --- | --- | --- |
| To what extent do you agree with each of the following statements? | Medications used at home can be harmful to children regardless of their type.  Children find it difficult to open medication packaging.  The responsibility for keeping medications out of children's reach lies with everyone in the household.  The optimal place to store household cleaners is under the kitchen sink.  Cleaners can be placed in visible locations as long as they are out of children's reach.  Household products like cleaners and cosmetics may contain toxic substances for children if not stored properly.  The safety information on household products is clear and easy to understand.  Social media is an important source of information.  The responsibility for educating children about the dangers of toxic household products lies with parents and caregivers. | Disagree  Neutral  Agree |

Table S4: Practices related to the safe use and storage of household products

| Do you use cleaning product containers to store something else? | Always Sometimes Never I don't remember |
| --- | --- |
| When was the last time you read the safety instructions for cleaning products? | Every time I use a new product When I think it might be dangerous I rarely read them Never I don't remember |
| Currently, where do you store household cleaners such as bathroom cleaners or laundry detergent in your home? (Select all that apply) | Under the kitchen sink (closed cabinet with a lock) Under the kitchen sink (open cabinet or without a lock) In the bathroom cabinet (closed with a lock) In the bathroom cabinet (open or without a lock) On a high shelf In a closed storage box On the kitchen or bathroom floor In another place (please specify) |
| Do you put household products away immediately after use? | Always Sometimes Never I don't remember |
| In the past year, how many times have you taught children at home about the dangers of household products? | Always Sometimes Never I don't remember |
| Have your children ever experienced a poisoning incident at home from cleaning products or medications? | Yes, it happened once  Yes, it happened multiple times  No, no poisoning has occurred  I don't remember |

Table S5: Association of scores of knowledge, attitude and practice (KAP) with the age of participants

| Group | Median % of maximum (quartiles) | | | | | p*-*value |
| --- | --- | --- | --- | --- | --- | --- |
|  | 18-25 (n= 123) | 26-35 (n =532) | 36-45 (n= 826) | 46-55 (n=382) | 56 and above (n =54) |  |
| Total KAP score | 59.6 (51.1, 67.0) | 61.7 (55.3, 69.1) | 62.8 (55.3, 69.1) | 61.7 (54.3, 68.1) | 63.3 (56.1, 68.1) | 0.024 |
| Total knowledge | 56.1 (45.5, 63.6) | 56.1 (48.5, 65.2) | 56.1 (48.5, 36.6) | 54.5 (45.5, 63.6) | 58.3 (48.1, 63.6) | 0.276 |
| Toxicity of household products | 61.5 (50.0, 76.9) | 65.4 (53.8, 76.9) | 65.4 (53.8, 76.9) | 65.4 (50.0, 76.9) | 65.4 (52.9, 76.9) | 0.828 |
| Toxicity of medications | 50.0 (35.7, 57.1) | 57.1(42.9, 65.4) | 57.1 (42.9, 64.3) | 57.1 (42.9, 64.3) | 57.1 (35.7, 66.1) | 0.144 |
| Corrosive substances in household products | 66.7 (50.0, 66.7) | 50.0 (50.0, 66.7) | 58.3 (50.0, 66.7) | 50.0 (50.0, 66.7) | 58.3 (50.0, 66.7) | 0.033* |
| Symptoms of poisoning | 57.1 (42.9, 85.7) | 57.1 (42.9, 85.7) | 57.1 (42.9, 85.7) | 57.1 (42.9, 75.0) | 71.4 (53.6, 89.3) | 0.048* |
| Poisoning management | 37.5 (0.0, 62.5) | 37.5 (0.0, 75.0) | 37.5 (0.0, 62.5) | 25.0 (0.0, 50.0) | 37.5 (0.0, 50.0) | 0.136 |
| Attitude | 72.2 (61.1, 83.3) | 77.8 (66.7, 83.3) | 77.8 (72.2, 88.9) | 77.8 (66.7, 83.3) | 77.8 (61.1, 84.7) | <0.001* |
| Practice | 70.0 (60.0, 90.0) | 80.0 (70.0, 90.0) | 80.0 (70.0, 90.0) | 80.0 (60.0, 90.0) | 80.0 (67.5, 90.0) | 0.002* |

* Statistically significant

Table S6: Association of scores of knowledge, attitude and practice (KAP) with region in the Kingdom of Saudi Arabia

| Group | Median % of maximum (quartiles) | | | | | p*-*value |
| --- | --- | --- | --- | --- | --- | --- |
|  | Northern (n =923) | Eastern (n =361) | Western (n =222) | Southern (n =128) | Central (n =283) |  |
| Total KAP score | 61.7 (54.3, 69.1) | 61.7 (55.3, 68.1) | 62.8 (56.4, 67.3) | 60.6 (53.2, 67.2) | 62.8 (55.3, 69.1) | 0.531 |
| Total knowledge | 56.1 (47.0, 63.6) | 56.0 (48.5, 63.6) | 57.6 (50.0, 640) | 53.8 (45.5, 62.1) | 57.6 (47.0, 65.2) | 0.47 |
| Toxicity of household products | 61.5 (50.0, 76.9) | 65.4 (54.8, 76.9) | 64.2 (57.7, 76.9) | 57.7 (50.0, 73.1) | 96.2 (53.8, 80.8) | <0.001* |
| Toxicity of medications | 57.1 (35.7, 64.3) | 57.1 (42.9, 64.3) | 57.1 (42.9, 64.3) | 50.0 (42.9, 57.1) | 57.1 (42.9, 64.3) | 0.265 |
| Corrosive substances in household products | 50.0 (50.0, 66.7) | 50.0 (50.0, 66.7) | 66,7 (50.0, 66.7) | 6.667 (50.0, 66.7) | 50.0 (50.0, 66.7) | 0.012* |
| Symptoms of poisoning | 57.1 (42.9, 71.4) | 71.4 (57.1, 85.7) | 71.4 (42.9, 85.7) | 71.4 (57.1, 85.7) | 57.1 (42.9, 71.3) | <0.001* |
| Poisoning management | 37.5 (0, 62.5) | 37.5 (0, 62.5) | 31.3 (0, 62.5) | 37.5 (0, 62.5) | 37.5 (0, 62.5) | 0.916 |
| Attitude | 77.8 (72.2, 88.9) | 77.8 (66.7, 83.3) | 77.8 (66.7, 83.3) | 77.8 (66.7, 83.3) | 77.8 (66.7, 83.3) | 0.002* |
| Practice | 80.0 (70.0, 90.0) | 80.0 (60.0, 90.0) | 70.0 (60.0, 90.0) | 80.0 (70.0, 90.0) | 80.0 (60.0, 90.0) | <0.001* |

* Statistically significant

Table S7: Association of scores of knowledge, attitude and practice (KAP) with the marital status of study participants

| Group | Median % of maximum (quartiles) | | | p*-*value |
| --- | --- | --- | --- | --- |
|  | Single (n =122) | Single parent (n =122) | Married (n =1673) |  |
| Total KAP score | 61.7 (53.2, 67.0) | 62.2 (53.2, 69.1) | 61.7 (55.3, 69.1) | 0.473 |
| Total knowledge | 57.6 (48.1, 63.6) | 57.8 (43.5, 64.0) | 56.1 (47.0, 63.6) | 0.932 |
| Toxicity of household products | 65.4 (53.8,76.9) | 65.4 (50.0, 76.9) | 65.4 (53.8, 78.9) | 0.892 |
| Toxicity of medications | 50.0 (35.7, 57.1) | 50.0 (41.1, 64.3) | 57.1 (42.9, 64.3) | 0.144 |
| Corrosive substances in household products | 66.7 (50.0,66.7) | 66.7 (50.0, 66.7) | 50.0 (50.0, 66.7) | 0.079 |
| Symptoms of poisoning | 71.4 (42.9, 85.7) | 57.1 (42.9, 85.7) | 37.1 (42.9, 85.7) | 0.382 |
| Poisoning management | 37.5 (9.4, 62.5) | 25.0 (0, 62.5) | 37.5 (0, 62.5) | 0.593 |
| Attitude | 77.8 (61.1, 83.3) | 77.8 (66.7, 88.9) | 77.8 (66.7, 83.3) | 0.477 |
| Practice | 70.0 (50.0, 90.0) | 90.0 (70.0, 100.0) | 80.0 (70.0, 90.0 | **<0.001*** |

* Statistically significant

Table S8: Association of scores of knowledge, attitude and practice (KAP) with the level of education of study participants

| Group | Median % of maximum (quartiles) | | | | | | p-value |
| --- | --- | --- | --- | --- | --- | --- | --- |
|  | No formal education (n=11) | Primary school (n=57) | Secondary school (n=72) | High school (n=395) | Graduate (n=1288) | Post-graduate (n=94) |  |
| Total KAP score | 54.3 (46.8, 56.4) | 57.4 (52.7, 64.9) | 57.4 (49.2, 65.7) | 61.7 (53.2, 69.1) | 62.8 (55.3, 69.1) | 64.9 (59.3, 69.4) | <0.001* |
| Total knowledge | 43.9 (37.9, 53.0) | 50.0 (40.9, 59.8) | 51.5 (40.9, 58.7) | 54.5 (45.5, 63.6) | 57.6 (48.5, 63.6) | 59.8 (54.2, 69.7) | <0.001* |
| Toxicity of household products | 53.8 (46.2, 69.2) | 57.7 (46.2, 69.2) | 57.7 (46.2, 73.1) | 61.5 (50.0, 76.9) | 65.4 (53.8, 76.9) | 73.1 (61.5, 80.8) | <0.001* |
| Toxicity of medications | 50.0 (21.4, 57.1) | 42.9 (28.6, 57.1) | 50.0 (28.6, 62.5) | 50.0 (35.7, 64.3) | 57.1 (42.9, 64.3) | 57.1 (48.2, 71.4) | <0.001* |
| Corrosive substances in household products | 50.0 (33.3, 66.7) | 50.0 (50.0, 66.7) | 50.0 (50.0, 66.7) | 50.0 (50.0, 66.7) | 50.0 (50.0, 66.7) | 66.7 (50.0, 66.7) | 0.597 |
| Symptoms of poisoning | 42.9 (14.3, 57.1) | 57.1 (42.9, 71.4) | 57.1 (17.9, 71.4) | 57.1 (28.6, 71.4) | 74.4 (42.9, 85.7) | 71.4 (57.1, 85.7) | <0.001* |
| Poisoning management | 12.5 (0.0, 50.0) | 37.5 (6.3, 62.5) | 25.0 (0.0, 59.4) | 37.5 (0.0, 75.0) | 37.5 (0.0, 62.5) | 37.5 (0.0, 65.6) | 0.212 |
| Attitude | 72.3 (33.3, 83.3) | 77.8 (69.4, 83.3) | 77.8 (61.6, 88.9) | 77.8 (66.7, 83.3) | 77.8 (66.7, 88.9) | 77.8 (66.7, 83.3) | 0.646 |
| Practice | 80.0 (50.0, 90.0) | 80.0 (60.0, 95.0) | 80.0 (70.0, 100.0) | 80.0 (70.0, 90.0) | 80.0 (70.0, 90.0) | 70.0 (60.0, 90.0) | 0.039* |

* Statistically significant

Table S9: Association of scores of knowledge, attitude and practice (KAP) with the employment status of study participants

| Group | Median % of maximum (quartiles) | | | | | | p*-*value |
| --- | --- | --- | --- | --- | --- | --- | --- |
|  | Unemployed  (n =800) | Student  (n =70) | Teacher/doctor  (n = 9) | Work outside the home (n = 923) | Retired (n = 79) | Works from home (n = 36) |  |
| Total KAP score | 61.7 (54.5, 69.1) | 61.7 (52.9, 67.0) | 65.9 (54.3, 69.1) | 62.8 (55.3, 69.1) | 61.7 (52.1,67.0) | 61.2 (54.3, 68.9) | 0.651 |
| Total knowledge | 56.1 (47.0, 63.6) | 57.6 (48.1, 65.2) | 59.1 (50.0, 62.9) | 56.1 (48.5, 63.6) | 56.1 (45.5, 63.6) | 56.8 (48.5, 65.2) | 0.735 |
| Toxicity of household products | 65.4 (50.0, 76.9) | 65.4 (53.8, 76.9) | 57.7 (46.2, 75.0) | 65.4 (53.8, 76.9) | 69.2 (53.8, 76.9) | 65.4 (50.0, 79.8) | 0.682 |
| Toxicity of medications | 50.0 (35.7, 64.3) | 50.0 (35.7, 58.9) | 57.1 (28.6, 71.4) | 57.1 (42.9, 64.3) | 57.1 (35.7, 64.3) | 57.1 (50.0, 69.6) | 0.006* |
| Corrosive substances in household products | 50.0 (50.0, 66.7) | 58.3 (50.0, 66.7) | 50.0 (50.0, 75.0) | 50.0 (50.0, 66.7) | 50.0 (50.0, 66.7) | 50.0 (50.0, 66.7) | 0.573 |
| Symptoms of poisoning | 57.1 (42.9, 85.7) | 71.4 (42.9, 85.7) | 57.1 (42.9, 85.7) | 57.1 (42.9, 85.7) | 57.1 (28.6, 71.4) | 71.4 (42.9, 71.4) | 0.61 |
| Poisoning management | 37.5 (0.0, 62.5) | 37.5 (9.4, 63.2) | 37.5 (0.0, 56.3) | 25.0 (0.0, 62.5) | 25.0 (0.0, 50.0) | 25.0 (0.0, 62.5) | 0.324 |
| Attitude | 77.8 (66.7, 88.9) | 72.2 (61.1, 83.3) | 77.8 (72.2, 83.3) | 77.8 (66.7, 88.9) | 77.8 (66.7, 83.3) | 72.2 (66.7, 83.3) | 0.127 |
| Practice | 80.0 (70.0, 90.0) | 70.0 (50.0, 82.5) | 90.0 (70.0, 100.0) | 80.0 (60.0, 90.0) | 80.6 (60.0, 90.0) | 80.0 (70.0, 90.0) | <0.001* |

* Statistically significant

Table S10: Association of scores of knowledge, attitude and practice (KAP) among healthcare and non-healthcare professionals

| Group | Median % of maximum (quartiles) | | p*-*value |
| --- | --- | --- | --- |
|  | Non-healthcare professional  (n = 1734) | Healthcare professional  (n = 183) |  |
| Total KAP score | 61.7 (54.6, 68.1) | 66.0 (58.5, 70.2) | <0.001* |
| Total knowledge | 56.1 (47.0, 63.6) | 62.1 (53.0, 68.2) | <0.001* |
| Toxicity of household products | 65.4 (50.0, 76.9) | 69.2 (53.8, 80.8) | 0.026* |
| Toxicity of medications | 57.1 (42.3, 64.3) | 57.1 (42.9, 64.3) | 0.113 |
| Corrosive substances in household products | 50.0 (50.0, 66.7) | 66.7 (50.0, 66.7) | 0.009* |
| Symptoms of poisoning | 57.1 (42.9, 71.4) | 71.4 (57.1, 85.7) | <0.001* |
| Poisoning management | 37.5 (0.0, 62.5) | 50.0 (0.0, 75.0) | <0.001* |
| Attitude | 77.8 (66.7, 88.9) | 77.8 (66.7, 83.3) | 0.206 |
| Practice | 80.0 (70.0, 90.0) | 80.0 (60.0, 90.0) | 0.008* |

* Statistically significant

Table S11: Association of scores of knowledge, attitude and practice (KAP) with the monthly income of study participants

| Group | Median % of maximum (quartiles) | | | | | p*-*value |
| --- | --- | --- | --- | --- | --- | --- |
|  | No income  (n = 580) | < 5000  (n =327) | 5000-10,000  (n =432) | 10,000-15,000  (n =379) | > 15,000  (n =199) |  |
| Total KAP score | 61.7 (54.2, 68.1) | 62.8 (54.3, 69.1) | 61.7 (55.3, 69.1) | 63.8 (56.4, 69.1) | 62.8 (57.4, 69.1) | 0.098 |
| Total knowledge | 54.5 (47.0, 62.1) | 56.1 (45.5, 63.6) | 56.1 (47.0, 65.2) | 57.6 (50.0, 63.6) | 57.6 (48.5, 65.2) | 0.005* |
| Toxicity of household products | 61.6 (50.0, 76.9) | 65.4 (50.0, 76.9) | 65.4 (53.8, 76.9) | 65.4 (53.8, 78.9) | 65.4 (53.8, 76.9) | 0.058 |
| Toxicity of medications | 50.0 (35.7, 64.3) | 50.0 (37.5, 64.3) | 57.1 (42.9, 64.3) | 57.1 (42.9, 64.3) | 57.1 (42.9, 64.3) | <0.001* |
| Corrosive substances in household products | 50.0 (50.0, 66.7) | 50.0 (50.0, 66.7) | 50.0 (50.0, 66.7) | 50.0 (50.0, 66.7) | 50.0 (50.0,66.7) | 0.759 |
| Symptoms of poisoning | 57.1 (42.9, 85.7) | 57.1 (42.9, 71.4) | 57.1 (42.9, 71.4) | 71.4 (57.1, 85.7) | 71.4 (42.9, 85.7) | <0.001* |
| Poisoning management | 37.5 (0.0, 62.5) | 37.5 (0.0, 75.0) | 37.5 (0.0, 62.5) | 25.0 (0.0,62.5) | 25.0 (0.0, 62.5) | 0.177 |
| Attitude | 77.8 (66.7, 88.9) | 77.8 (66.7, 83.3) | 77.8 (66.7, 83.3) | 77.8 (66.7, 88.9) | 77.8 (72.2, 83.3) | 0.621 |
| Practice | 80.0 (70.0, 90.0) | 80.0 (70.0, 90.0) | 80.0 (70.0, 90.0) | 80.0 (60.0, 90.0) | 80.0 (60.0, 90.0) | 0.031* |

* Statistically significant

Table S12: Association of scores of knowledge, attitude and practice (KAP) with the relation of study participants to children

| Group | Median % of maximum (quartiles) | | | | | p*-*value |
| --- | --- | --- | --- | --- | --- | --- |
|  | Mother  (n = 1454) | Father  (n = 256) | Grandparent  (n = 65) | Sibling  (n = 78) | Other  (n = 63) |  |
| Total KAP score | 62.8 (55.3, 69.1) | 59.6 (52.1, 67.0) | 64.9 (57.4, 69.1) | 60.6 (51.9, 67.0) | 61.7 (56.4, 68.1) | 0.002* |
| Total knowledge | 56.1 (48.5, 63.6) | 51.5 (42.4, 62.1) | 57.6 (51.5, 65.2) | 56.8 (47.0, 64.0) | 57.6 (50.0, 62.1) | 0.001* |
| Toxicity of household products | 65.4 (53.8, 76.9) | 61.5 (50.0, 73.1) | 65.4 (61.5, 76.9) | 69.2 (57.7, 76.9) | 69.2 (53.8, 76.9) | <0.001* |
| Toxicity of medications | 57.1 (42.9, 64.3) | 50.5 (35.7, 64.3) | 57.1 (42.9, 64.3) | 50.0 (33.9, 57.1) | 57.1 (42.9, 71.4) | 0.016* |
| Corrosive substances in household products | 50.0 (50.0, 66.7) | 50.0 (50.0, 66.7) | 66.7 (50.0, 66.7) | 66.7 (50.0, 66.7) | 50.0 (50.0, 66.7) | 0.066 |
| Symptoms of poisoning | 57.1 (42.9, 85.7) | 57.1 (41.3, 71.4) | 71.4 (42.3, 85.7) | 71.4 (53.6, 85.7) | 57.1 (42.9, 85.7) | <0.001* |
| Poisoning management | 37.5 (0.0, 62.5) | 26.0 (0.0, 62.5) | 37.5 (0.0, 62.5) | 25.0 (0.0, 62.5) | 37.5 (0.0, 62.5) | 0.037* |
| Attitude | 77.8 (66.7, 88.9) | 77.8 (66.7, 83.3) | 77.8 (66.7, 86.1) | 77.8 (61.1, 83.3) | 77.8 (66.7, 88.9) | 0.534 |
| Practice | 80.0 (70.0, 90.0) | 80.0 (60.0, 90.0) | 80.0 (60.0, 90.0) | 75.0 (50.0, 90.0) | 70.0 (60.0, 90.0) | <0.001* |

* Statistically significant

Table S13: Association of scores of knowledge, attitude and practice (KAP) with the number of children in the household

| Group | Median % of maximum (quartiles) | | | | | p*-*value |
| --- | --- | --- | --- | --- | --- | --- |
|  | 1 (n = 436) | 2 (n = 566) | 3 (n = 438) | 4 (n = 270) | 5-10 (n = 207) |  |
| Total KAP score | 60.6 (54.3, 68.1) | 61.7 (55.3, 68.1) | 63.8 (57.2, 69.1) | 61.7 (55.3, 68.1) | 60.4 (54.3, 68.1) | 0.014* |
| Total knowledge | 54.5 (45.4, 63.6) | 56.1 (48.5, 63.6) | 57.6 (50.0, 65.2) | 54.5 (47.0, 63.6) | 54.5 (44.0, 63.6) | 0.001* |
| Toxicity of household products | 65.4 (51.0, 76.9) | 65.4 (53.8, 76.9) | 65.4 (53.8, 76.9) | 65.4 (50.0, 76.9) | 61.5 (50.0, 79.9) | 0.139 |
| Toxicity of medications | 50.0 (37.5, 37.1) | 57.1 (42.9, 64.3) | 57.1 (42.9, 64.3) | 57.1 (42.9, 64.3) | 57.1 (35.7, 64.3) | 0.101 |
| Corrosive substances in household products | 50.0 (50.0, 66.7) | 66.7 (50.0, 66.7) | 50.0 (50.0, 66.7) | 50.0 (50.0, 66.7) | 50.0 (50.0, 66.7) | 0.09 |
| Symptoms of poisoning | 57.1 (42.9, 85.7) | 71.4 (42.9, 85.7) | 64.3 (42.9, 85.7) | 57.1 (42.9, 71.4) | 57.1 (28.6, 71.4) | 0.015* |
| Poisoning management | 37.5 (0.0, 62.5) | 37.5 (0.0, 62.5) | 37.5 (0.0, 62.5) | 25.0 (0.0, 62.5) | 25.0 (0.0, 62.5) | 0.356 |
| Attitude | 77.8 (66.7, 83.3) | 77.8 (66.7, 83.3) | 77.8 (66.7, 88.9) | 77.8 (72.2, 88.9) | 77.8 (66.7, 88.9) | 0.129 |
| Practice | 80.0 (60.0, 90.0) | 80.0 (60.0, 90.0) | 80.0 (70.0, 90.0) | 80.0 (70.0, 90.0) | 80.0 (70.0, 90.0) | 0.057 |

* Statistically significant

Table S14: Association of scores of knowledge, attitude and practice (KAP) with the age of children in the household

| Group | Median % of maximum (quartiles) | | p*-*value |
| --- | --- | --- | --- |
|  | No children ≤5 years (n =779) | Children ≤5 years (n =1138) |  |
| Total KAP score | 61.7 (55.3, 68.1) | 62.8 (54.2, 69.1) | 0.365 |
| Total knowledge | 56.1 (47.0, 63.6) | 65.1 (47.0, 63.6) | 0.371 |
| Toxicity of household products | 65.4 (50.0, 76.9) | 65.4 (53.8, 76.9) | 0.083 |
| Toxicity of medications | 57.1 (42.9, 64.3) | 57.1 (42.9, 64.3) | 0.113 |
| Corrosive substances in household products | 50.0 (50.0, 66.7) | 50.0 (50.0, 66.7) | 0.155 |
| Symptoms of poisoning | 57.1 (42.9, 85.7) | 57.1 (42.9, 85.7) | 0.435 |
| Poisoning management | 37.5 (0.0, 62.5) | 37.5 (0.0, 62.5) | 0.283 |
| Attitude | 77.8 (66.7, 83.3) | 77.8 (66.7, 88.9) | 0.504 |
| Practice | 80.0 (70.0, 90.0) | 80.0 (70.0, 90.0) | 0.701 |

Table S15: Association of scores of knowledge, attitude and practice (KAP) with the presence of a domestic helper in the household

| Group | Median % of maximum (quartiles) | | p*-*value |
| --- | --- | --- | --- |
|  | No housemaid  (n =1064) | ≥1 housemaid  (n = 853) |  |
| Total KAP score | 61.7 (54.3, 69.1) | 62.7 (56.4, 69.1) | 0.137 |
| Total knowledge | 54.5 (45.5, 63.6) | 57.6 (48.5, 63.6) | 0.001* |
| Toxicity of household products | 65.4 (50.9, 76.9) | 65.4 (53.8, 76.9) | 0.004* |
| Toxicity of medications | 57.1 (35.7, 64.3) | 57.1 (42.9, 64.3) | 0.022* |
| Corrosive substances in household products | 50.0 (50.0, 66.7) | 50.0 (50.0, 66.7) | 0.015* |
| Symptoms of poisoning | 57.1 (42.9, 82.1) | 71.4 (42.9, 85.7) | 0.005* |
| Poisoning management | 37.5 (0.0, 62.5) | 25.0 (0.0, 62.5) | 0.095 |
| Attitude | 77.8 (66.7, 83.3) | 77.8 (66.7, 83.3) | 0.689 |
| Practice | 80.0 (70.0, 90.0) | 80.0 (60.0, 90.0) | <0.001* |

* Statistically significant

Table S16: Total KAP satisfactory score regression analysis

| **Forced entry model** | **OR** | **95% C.I. for OR** | | **p*-*value** |
| --- | --- | --- | --- | --- |
|  |  | **Lower** | **Upper** |  |
| Sex (male) | 0.531 | 0.282 | 0.998 | **0.049*** |
| Employment status (category) | 0.935 | 0.645 | 1.355 | 0.724 |
| Healthcare profession | 1.964 | 1.368 | 2.821 | **<0.001*** |
| Monthly income over 10,000 SR | 1.153 | 0.916 | 1.450 | 0.225 |
| Mother | 1.020 | 0.666 | 1.562 | 0.927 |
| Father | 1.141 | 0.541 | 2.407 | 0.729 |
| Grandparent | 1.546 | 0.771 | 3.098 | 0.220 |
| Age below 25 | 0.627 | 0.398 | 0.987 | 0.044* |
| Household with child ≤5 years | 0.992 | 0.808 | 1.219 | 0.941 |
| Households with at least one domestic helper | 0.986 | 0.808 | 1.203 | 0.889 |
| Household with ≥3 children | 1.263 | 1.034 | 1.543 | 0.022* |
| Education (category) | 1.249 | 1.103 | 1.415 | <0.001* |

* Statistically significant; Employment status categories were coded from 1 to 6 for: unemployed, student, teacher/doctor, work outside the home, retired works from home, respectively; Education categories were coded from 1 to 6 for: No formal education, primary school, secondary school, graduate, post-graduate, respectively

Table S17: Knowledge satisfactory score regression analysis

| **Forced entry model** | **OR** | **95% C.I. for OR** | | **p*-*value** |
| --- | --- | --- | --- | --- |
|  |  | **Lower** | **Upper** |  |
| Sex (male) | 0.526 | 0.267 | 1.037 | 0.064 |
| Employment status (category) | 0.870 | 0.595 | 1.270 | 0.470 |
| Healthcare profession | 2.000 | 1.437 | 2.784 | <0.001 |
| Monthly income over 10,000 SR | 1.036 | 0.823 | 1.304 | 0.761 |
| Mother | 1.240 | 0.798 | 1.928 | 0.339 |
| Father | 1.700 | 0.761 | 3.797 | 0.196 |
| Grandparent | 1.624 | 0.822 | 3.210 | 0.163 |
| Age below 25 years | 0.860 | 0.533 | 1.387 | 0.536 |
| Household with child ≤5 years | 0.992 | 0.803 | 1.225 | 0.937 |
| Households with at least one domestic helper | 1.142 | 0.933 | 1.398 | 0.199 |
| Household with ≥3 children | 1.252 | 1.021 | 1.536 | 0.031 |
| Education (category) | 1.247 | 1.089 | 1.429 | 0.001 |

Employment status categories were coded from 1 to 6 for: unemployed, student, teacher/doctor, work outside the home, retired works from home, respectively; Education categories were coded from 1 to 6 for: No formal education, primary school, secondary school, graduate, post-graduate, respectively

Table S18: Attitude satisfactory score regression analysis

| **Forced entry model** | **OR** | **95% C.I. for OR** | | **p*-*value** |
| --- | --- | --- | --- | --- |
|  |  | **Lower** | **Upper** |  |
| Sex (male) | 2.081 | 0.764 | 5.664 | 0.152 |
| Employment status (category) | 1.203 | 0.736 | 1.968 | 0.461 |
| Healthcare profession | 0.970 | 0.609 | 1.547 | 0.900 |
| Monthly income over 10,000 SR | 0.979 | 0.706 | 1.358 | 0.898 |
| Mother | 0.885 | 0.482 | 1.626 | 0.694 |
| Father | 0.320 | 0.100 | 1.018 | 0.054 |
| Grandparent | 0.958 | 0.354 | 2.588 | 0.932 |
| Age below 25 years | 0.521 | 0.294 | 0.923 | 0.025 |
| Household with child ≤5 years | 0.986 | 0.735 | 1.324 | 0.926 |
| Households with at least one domestic helper | 0.935 | 0.704 | 1.242 | 0.644 |
| Household with ≥3 children | 1.047 | 0.787 | 1.394 | 0.751 |
| Education (category) | 1.109 | 0.937 | 1.314 | 0.230 |

Employment status categories were coded from 1 to 6 for: unemployed, student, teacher/doctor, work outside the home, retired works from home, respectively; Education categories were coded from 1 to 6 for: No formal education, primary school, secondary school, high school, graduate, post-graduate, respectively

Table S19: Practice satisfactory score regression analysis

| **Forced entry model** | **OR** | **95% C.I. for OR** | | **p*-*value** |
| --- | --- | --- | --- | --- |
|  |  | **Lower** | **Upper** |  |
| Sex (male) | 1.507 | .645 | 3.517 | 0.343 |
| Employment status (category) | 1.241 | 0.765 | 2.015 | 0.382 |
| Healthcare profession | 0.940 | 0.592 | 1.494 | 0.794 |
| Monthly income over 10,000 SR | 0.836 | 0.601 | 1.163 | 0.287 |
| Mother | 2.207 | 1.291 | 3.772 | 0.004 |
| Father | 0.823 | 0.309 | 2.191 | 0.696 |
| Grandparent | 1.037 | 0.457 | 2.349 | 0.931 |
| Age below 25 years | 0.696 | 0.383 | 1.268 | 0.237 |
| Household with child ≤5 years | 0.952 | 0.703 | 1.289 | 0.749 |
| Households with at least one domestic helper | 0.733 | 0.546 | 0.982 | 0.038 |
| Household with ≥3 children | 1.257 | 0.934 | 1.690 | 0.131 |
| Education (category) | 1.135 | 0.948 | 1.359 | 0.168 |

Employment status categories were coded from 1 to 6 for: unemployed, student, teacher/doctor, work outside the home, retired works from home, respectively; Education categories were coded from 1 to 6 for: No formal education, primary school, secondary school, high school, graduate, post-graduate, respectively

Table S20: Total KAP percentage score — linear regression sensitivity analysis

| **Variable** | **β (coef.)** | **95% CI** | | | **p-value** |
| --- | --- | --- | --- | --- | --- |
|  |  | **Lower** | **Upper** | |  |
| Sex (male) | -2.195 | -6.146 | | 1.756 | 0.276 |
| Employment status | -0.221 | -0.524 | | 0.082 | 0.153 |
| Healthcare-related profession | 3.351 | 1.576 | | 5.126 | <0.001* |
| Monthly income >10,000 SR | 0.606 | -0.586 | | 1.797 | 0.319 |
| Mother | -0.379 | -2.553 | | 1.795 | 0.733 |
| Father | -1.034 | -5.699 | | 3.631 | 0.664 |
| Grandparent | 1.401 | -1.805 | | 4.608 | 0.392 |
| Age <25 years | -3.561 | -6.236 | | -0.886 | 0.009* |
| Household with child ≤5 years | -0.307 | -1.352 | | 0.738 | 0.565 |
| Household with domestic helper | 0.337 | -0.719 | | 1.393 | 0.531 |
| Households with ≥3 children | 1.144 | 0.134 | | 2.154 | 0.026* |
| Higher educational level | 1.674 | 0.929 | | 2.418 | <0.001* |

Table S21: Knowledge percentage score — linear regression sensitivity analysis

| **Variable** | **β (coef.)** | **95% CI** | | | **p-value** |
| --- | --- | --- | --- | --- | --- |
|  |  | **Lower** | | **Upper** |  |
| Sex (male) | -3.662 | -8.020 | 0.695 | | 0.100 |
| Employment status | -0.162 | -0.524 | 0.200 | | 0.381 |
| Healthcare-related profession | 5.149 | 3.122 | 7.176 | | <0.001* |
| Monthly income >10,000 SR | 0.693 | -0.703 | 2.088 | | 0.331 |
| Mother | -0.862 | -3.295 | 1.572 | | 0.488 |
| Father | -0.772 | -5.910 | 4.366 | | 0.768 |
| Grandparent | 2.159 | -1.397 | 5.715 | | 0.234 |
| Age <25 years | -2.610 | -5.699 | 0.479 | | 0.098 |
| Household with child ≤5 years | -0.372 | -1.608 | 0.864 | | 0.555 |
| Household with domestic helper | 0.861 | -0.398 | 2.120 | | 0.180 |
| Households with ≥3 children | 1.094 | -0.108 | 2.295 | | 0.074 |
| Higher educational level | 2.186 | 1.349 | 3.024 | | <0.001* |

Table S22: Attitude percentage score — linear regression sensitivity analysis

| **Variable** | **β (coef.)** | **95% CI** | | | **p-value** |
| --- | --- | --- | --- | --- | --- |
|  |  | **Lower** | | **Upper** |  |
| Sex (male) | 0.811 | -5.046 | 6.668 | | 0.786 |
| Employment status | -0.371 | -0.834 | 0.093 | | 0.117 |
| Healthcare-related profession | -0.800 | -3.486 | 1.885 | | 0.559 |
| Monthly income >10,000 SR | 0.871 | -1.030 | 2.771 | | 0.369 |
| Mother | -1.017 | -4.608 | 2.574 | | 0.579 |
| Father | -2.141 | -9.116 | 4.834 | | 0.547 |
| Grandparent | -0.633 | -5.810 | 4.543 | | 0.811 |
| Age <25 years | -5.918 | -10.021 | -1.814 | | 0.005* |
| Household with child ≤5 years | 0.082 | -1.585 | 1.749 | | 0.923 |
| Household with domestic helper | -0.120 | -1.745 | 1.506 | | 0.885 |
| Households with ≥3 children | 0.702 | -0.896 | 2.301 | | 0.389 |
| Higher educational level | 0.939 | -0.209 | 2.088 | | 0.109 |

Table S23: Practice percentage score — linear regression sensitivity analysis

| **Variable** | **β (coef.)** | **95% CI** | | | **p-value** |
| --- | --- | --- | --- | --- | --- |
|  |  | **Lower** | | **Upper** |  |
| Sex (male) | 2.080 | -3.440 | 7.600 | | 0.460 |
| Employment status | -0.342 | -0.874 | 0.191 | | 0.208 |
| Healthcare-related profession | -1.044 | -4.090 | 2.003 | | 0.502 |
| Monthly income >10,000 SR | -0.445 | -2.684 | 1.794 | | 0.697 |
| Mother | 3.956 | -0.166 | 8.077 | | 0.060 |
| Father | -0.773 | -7.559 | 6.014 | | 0.823 |
| Grandparent | 0.061 | -6.281 | 6.403 | | 0.985 |
| Age <25 years | -5.600 | -9.814 | -1.385 | | 0.009* |
| Household with child ≤5 years | -0.578 | -2.435 | 1.279 | | 0.542 |
| Household with domestic helper | -2.294 | -4.218 | -0.371 | | 0.019* |
| Households with ≥3 children | 2.274 | 0.484 | 4.064 | | 0.013* |
| Higher educational level | -0.386 | -1.610 | 0.838 | | 0.537 |

Table S24: Full demographic characteristics of study participants (non-collapsed categories; superseded Table 1)

| Characteristic | Number (%) |
| --- | --- |
| Region  Northern  Southern  Western  Eastern  Central | 923 (48.1)  128 (6.7)  222 (11.6)  361 (18.8)  283 (14.8) |
| Age (years)  18–25  26–35  36–45  46–55  ≥56 | 123 (6.4)  532 (27.8)  826 (43.1)  382 (19.9)  54 (2.8) |
| Sex  Male  Female | 295 (15.4)  1,622 (84.6) |
| Marital status  Single  Single-parent family (separated or widowed)  Married | 122 (6.4)  122 (6.4)  1,673 (87.3) |
| Highest level of education  No formal education  Primary school  Secondary school  High school  Graduate (diploma or bachelor)  Postgraduate (master, doctorate, or other) | 11 (0.6)  57 (3.0)  72 (3.8)  395 (20.6)  1,288 (67.2)  94 (4.9) |
| Employment status  Employed (work outside the home)  Employed (work from home)  Unemployed/Homemaker  Student  Retired | 932 (48.6)  36 (1.9)  800 (41.7)  70 (3.7)  79 (4.1) |
| Healthcare-related professions (e.g., nurse, doctor, and pharmacist)  No  Yes | 1,734 (90.5)  183 (9.5) |
| Monthly income (SAR)  <5,000  5,000–10,000  11,000–15,000  >15,000  No income | 327 (17.1)  432 (22.5)  379 (19.8)  199 (10.4)  580 (30.3) |
| Relation to children  Mother  Father  Grandparent  Siblings  Others (e.g., stepmother) | 1,455 (75.8)  256 (13.4)  65 (3.4)  78 (4.1)  63 (3.3) |
| Number of children (<12 years)  1  2  3  4  5–10 | 436 (22.7)  566 (29.5)  438 (22.8)  270 (14.1)  207 (10.8) |
| Age of children (years) *  ≤1  2–3  4–5  6–7  8–9  10–12 | 326 (17.0)  560 (29.2)  693 (36.2)  751 (39.2)  858 (44.8)  1,014 (52.9) |
| Number of domestic helpers  None  1  2  >3 | 1,064 (55.5)  624 (32.6)  73 (3.8)  156 (8.1) |
